# Editorial: Research on the correlative mechanisms and clinical exploration of headache and cerebrovascular diseases

**DOI:** 10.3389/fnins.2026.1848705

**Published:** 2026-05-13

**Authors:** Wei Wang, Feng Liu, Wei Shan, Lanfranco Pellesi

**Affiliations:** 1 Department of Neurology, Sir Run Run Shaw Hospital, School of Medicine, Zhejiang University, Hangzhou, China; 2 Department of Radiology and Tianjin Key Laboratory of Functional Imaging and Tianjin Institute of Radiology, Tianjin Medical University General Hospital, Tianjin, China; 3 Department of Neurology, Beijing Tiantan Hospital, Capital Medical University, Beijing, China; 4 National Center for Clinical Medicine of Neurological Diseases, Beijing, China; 5 Clinical Pharmacology, Pharmacy and Environmental Medicine, Department of Public Health, University of Southern Denmark, Odense, Denmark

**Keywords:** cerebrovascular diseases, clinical biomarkers, deep learning, endovascular treatment, headache disorders, neuroimaging

## Introduction

Headache disorders and cerebrovascular diseases have long been managed as distinct disciplines within clinical neurology. However, evolving epidemiological data, coupled with advanced neuroimaging and molecular profiling, have illuminated a bidirectional interplay between these two global health burdens. Severe headache syndromes, particularly migraine and thunderclap headache, can no longer be viewed merely as isolated, debilitating sensory experiences. Instead, they are increasingly recognized as phenotypic expressions of underlying cerebrovascular vulnerabilities.

The pathophysiological continuum connecting headache disorders and cerebrovascular diseases involves a complex cascade: endothelial dysfunction, neurovascular uncoupling, shared genetic susceptibility and aberrant inflammatory responses. Despite these conceptual leaps, translating this knowledge into precise diagnostic criteria and targeted therapeutics remains a challenge. The precise molecular pathways bridging ischemic stroke, intracranial aneurysms and severe headache syndromes remain incompletely defined. To address this knowledge gap, this Research Topic was curated to review and expand upon the multifaceted interactions within the neurovascular ecosystem. Spanning 19 contributions, this Research Topic traverses the cutting-edge landscapes of single-cell transcriptomics, multimodal artificial intelligence, dynamic neuroimaging and innovative interventional techniques. Together, these studies provide a

comprehensive theoretical and practical framework that enhances our understanding of headaches and cerebrovascular diseases.

## Molecular mechanisms and systemic biomarkers

Utilizing single-cell RNA sequencing (scRNA-seq) integrated with genome-wide association study (GWAS) data, Deng et al. mapped the high-resolution cellular landscape of large artery atherosclerotic stroke. Their analysis identified two distinct endothelial cell subtypes (aEC_cluster and EC2_cluster) and elucidated how genetic risk loci dictate specific cellular expression profiles through inflammatory signaling pathways, particularly those involving the transcriptional regulators Fos and Nr4a1. Translating molecular vulnerability to the clinical bedside, Yan and Lu engineered a highly discriminatory predictive model utilizing a peripheral blood biomarker panel. By integrating the platelet-to-lymphocyte ratio (PLR), lipoprotein-associated phospholipase A2 (Lp-PLA2), monocyte-to-high-density lipoprotein ratio (MHR) and the systemic immune-inflammation index (SII), they developed a dynamic nomogram to stratify the rupture risk of small to medium-sized intracranial aneurysms. Further confirming the role of systemic metabolic indices in stroke recovery, Zhu, Bi et al. conducted a multicenter study demonstrating a significant association between hemoglobin levels and balance function rehabilitation, emphasizing the necessity of holistic physiological monitoring in cerebrovascular disease management.

## The deep learning revolution and multimodal diagnostics

The integration of Artificial Intelligence (AI), specifically Deep Learning (DL), represents a transformative leap in neurovascular assessment. A comprehensive review by Mao et al. details this technological evolution, demonstrating how advanced DL architectures such as U-Net and DeepMedic are achieving expert-level diagnostic accuracy in the automated segmentation of intra- and extracranial arteries, as well as the precise quantification of plaque burden. Pushing this AI paradigm into the realm of multimodal integration, Zhao et al. developed NeuroFusionNet. This pioneering framework combines a ResNet-based visual encoder for complex medical image analysis with a multilayer perceptron branch strictly dedicated to interpreting clinical indicators. Achieving an accuracy of 0.9655 in the binary classification of cerebral infarction, this study proves that fusing structural imaging with systemic clinical biomarkers enhances diagnostic sensitivity. The prognostic application of these computational frameworks is illustrated by two additional studies. Li, Chen et al. utilized a DenseNet-based architecture to calculate “brain age” from unenhanced MRI scans in patients with Moyamoya disease. They found that accelerated brain aging correlates directly with the severity of arterial stenosis, positioning AI-derived brain age as a quantifiable biomarker for cumulative disease burden. Meanwhile, Zhang, Jin et al. developed a neurologist-level interpretable CT-based deep neural network that reliably predicts hemorrhagic transformation following ischemic stroke, providing a transparent tool for acute risk management.

## Clinical phenotyping, neuroimaging, and headache as a sentinel marker

Advanced neuroimaging continues to unravel complex, subjective pain mechanisms. Wang et al. reviewed the profound impact of chronic pain in medication overuse headache (MOH), detailing the maladaptive structural and functional plasticity within the nociceptive pain matrix and reward circuitry. Specifically, they highlighted that these neurobiological changes are partially reversible following medication withdrawal. In the realm of differential diagnosis, Zhang, Wang et al. utilized videonystagmography and caloric testing to successfully differentiate vestibular migraine from Meniere's disease based on objective physiological biomarkers.

Recognizing specific headache phenotypes as acute precursors to cerebrovascular catastrophes is a vital diagnostic skill. Zhang, He et al. provided a detailed mini-review on reversible cerebral vasoconstriction syndrome (RCVS), emphasizing the dangerous temporal dissociation wherein the peak of ischemic risk often lags days or weeks behind the initial thunderclap headache. Directly bridging the two pillars of this Research Topic, Yao et al. contributed a comprehensive review on post-stroke headache (PSH), elucidating its epidemiology and pathophysiology.

Furthermore, altered cerebrospinal fluid dynamics present neurovascular risks. Yang, Zhang et al. reported on clinical insights regarding intracranial hypotension headache complicated by retroclival subdural hematoma, showcasing how mechanical traction impacts fragile venous structures. Finally, focusing on alternative therapies, Zhang, Ouyang et al. utilized functional neuroimaging to localize brain networks activated by acupuncture, offering a neurobiological foundation for non-pharmacological neuromodulation in both migraine and stroke rehabilitation.

## Endovascular, surgical, and environmental interventions

Translating pathophysiological insights into tangible therapeutic benefits remains the ultimate objective of this Research Topic. In acute ischemic stroke management, Chen et al. demonstrated that a complete anterior Circle of Willis significantly enhances the aspiration efficacy of balloon guide catheters (BGC) during mechanical thrombectomy for internal carotid artery occlusions, resulting in higher first-pass recanalization rates. For symptomatic intracranial atherosclerotic stenosis (ICAS), Sun et al. conducted a comparative study revealing that drug-coated balloon (DCB) predilation significantly curtails intimal hyperplasia, reducing the incidence of in-stent restenosis compared to conventional balloons.

Addressing anatomical anomalies, a multicenter retrospective analysis by Yifei et al. identified that a smaller nidus size and a larger flow-related aneurysm size ratio independently predict the rupture of posterior circulation arteriovenous malformations (AVMs), providing insights for prophylactic surgical planning. In the context of hemorrhagic stroke, Yang, Huang et al. confirmed through a matched cohort study that proactive surgical evacuation for spontaneous cerebellar hemorrhages exceeding 10 mL serves as an independent predictor of favorable short-term functional outcomes and diminished mortality.

Beyond intrinsic vascular pathology, environmental triggers and pharmacological interactions play critical roles in acute events. Li, Gao et al. documented a rare case of acute intraventricular hemorrhage triggered by severe coughing following a return from high altitude, highlighting how extreme environmental hemodynamic shifts compromise fragile neurovascular structures. Lastly, Zhu, Chen et al. brought attention to neurocritical care pharmacology via a case report. They documented severe, drug-induced thrombocytopenia triggered by the concomitant administration of tirofiban (for stroke progression) and ibuprofen (for concomitant headache), advocating for hematological monitoring when managing overlapping treatment paradigms.

## Conclusion

The complex intersection of headache disorders and cerebrovascular diseases is emerging as one of the most dynamic, consequential frontiers in modern neurology. The 19 articles compiled in this Research Topic represent a leap forward for our discipline. By weaving together the threads of single-cell transcriptomic vulnerabilities, multi-modal AI-driven diagnostics, dynamic clinical phenotyping and refined neuro-interventional techniques, this Research Topic frames headaches not as isolated sensory complaints, but as integral components of the broader neurovascular ecosystem.

As we look toward the future, the continued integration of deep learning algorithms with multi-omics data and systemic clinical biomarkers promises to yield unprecedented predictive accuracy. We extend our deepest, most sincere gratitude to the contributing authors for their scholarship, the rigorous peer reviewers for their critical insights, and the editorial team at Frontiers for their unwavering support. It is our profound hope that these findings will catalyze sustained interdisciplinary collaboration, ultimately reshaping clinical guidelines and enhancing precision medicine strategies for our patients worldwide.

